# A Rapidly Enlarging Skin Lump

**DOI:** 10.7759/cureus.49566

**Published:** 2023-11-28

**Authors:** Melroy Rasquinha, Jason Adhikaree

**Affiliations:** 1 Otolaryngology, Nottingham University Hospital National Health Service (NHS) Trust, Nottingham, GBR; 2 Oncology, Nottingham University Hospital National Health Service (NHS) Trust, Nottingham, GBR

**Keywords:** radio-immunotherapy, squamous cell lung carcinoma, primary lung, painless lump, cutaneous malignancy

## Abstract

Lung cancer is one of the most common types of malignancy in the world associated with poor prognosis and an overall five-year survival rate of around 15%. Frequent sites of metastasis are the liver, brain, adrenal glands, hilar nodes, and bone. Metastasis to the skin is uncommon with an occurrence rate of 0.7%-9%. We report here an interesting case of an elderly woman who presented with a rapidly growing, substantially large fungating neck lump that turned out to be a cutaneous metastasis neck secondary to squamous cell carcinoma of the lungs.

## Introduction

Lung cancer is one of the most common types of malignancy in the world, associated with poor prognosis and an overall five-year survival rate of around 15%. Frequent sites of metastasis are the liver, brain, adrenal glands, hilar nodes, and bone [1]. Metastasis to the skin is uncommon with an occurrence rate of 0.7%-9%^ ^[2]. We report here a case of an elderly woman who presented with a rapidly growing, substantially large fungating neck lump which was later revealed to be a cutaneous metastasis secondary to squamous cell carcinoma of the lungs.

## Case presentation

An 81-year-old female with a background of hypertension, cervical spondylosis, and chronic obstructive pulmonary disease presented in the outpatient setting with a three to four-week history of a 2.5 cm lower midline neck lump. She denied any allergies and was independent with activities of daily living. Social, occupational, and family history were all unremarkable with having given up smoking over 20 years ago. Other than reporting mild lethargy she was asymptomatic. An ultrasound scan was found to be in keeping with an infectious sebaceous cyst and she was discharged with oral antibiotics.

**Figure 1:**
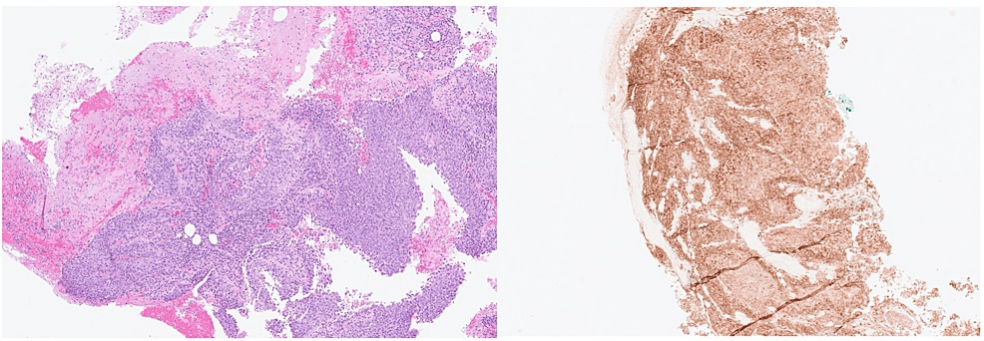
The biopsy images demonstrate clusters and sheets of atypical squamous cells (left) and when tested with IHC assay, PD-L1 staining (right) IHC: Immunohistochemistry

**Figure 2:**
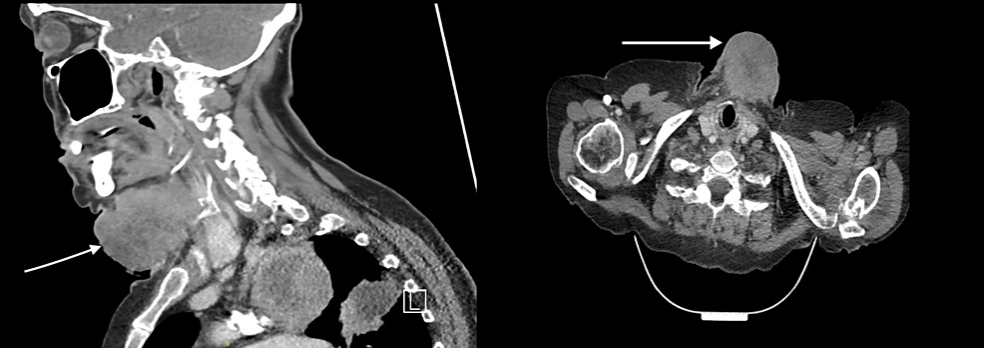
Computed tomography demonstrating large anterior neck subcutaneous deposit along with a lung primary (left: sagittal view, right: axial view)

The patient represented two weeks later with an increase in lump size, associated with pain and blood oozing from the mass which was estimated at around 500 ml. On examination, the lump demonstrated a fungating ulcerated appearance associated with a foul-smelling discharge. There was no craniofacial or cervical lymphadenopathy on examination and the oral cavity and flexible nasendoscopy were unremarkable. Paraprotein and virology screen were negative. A sample sent for analysis was reported to be negative for microscopy, culture and sensitivity, and acid-fast bacilli was not seen. Histology confirmed squamous cell carcinoma with PD-L1 almost 90% (Figure 1). AE1/3, CK7, and P40 were also found to be positive. The tumor cells were found to be negative for CK20, TTF1, CD56, and p16 on immunohistochemistry. Cross-sectional imaging of the neck, thorax, and abdomen reported an anterior soft tissue mass measuring 6.1 cm, left upper lobe mass, and right lung metastases along with a subcutaneous metastatic deposit in the neck (Figure 2).

The outcome of the pulmonology multi-disciplinary meeting was for surgical intervention in the first instance. However, surgery was deemed to be too extensive by the plastic surgery team; hence, an oncology opinion was sought.

**Figure 3:**
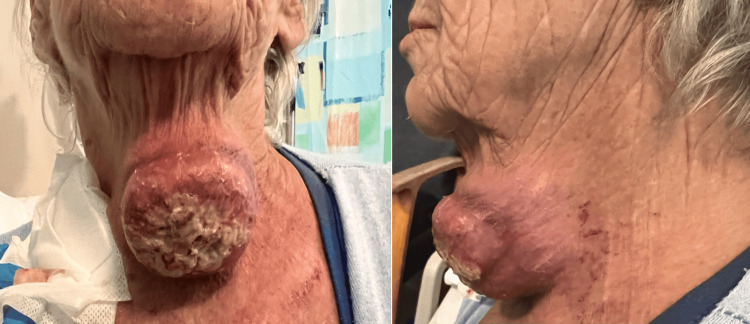
Antero-posterior (left) and lateral view (right) of an isolated fungating ulcerated anterior neck mass measuring approximately 10 x 8 cm

**Figure 4:**
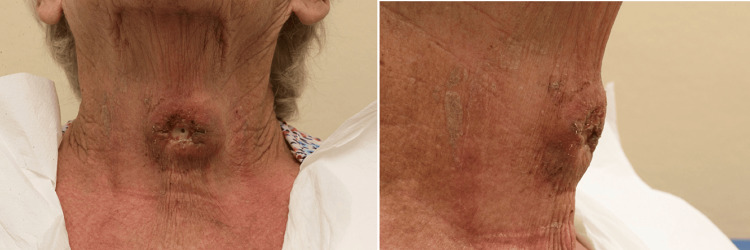
Antero-posterior (left) and lateral view (right) demonstrating the effects after one cycle of radio-immunotherapy

The decision was made to commence the patient on palliative radiotherapy followed by Pembrolizumab immunotherapy. By initiation of treatment, the lump had grown to be 10 cm in size and protruding 8 cm outwards from the neck (Figure 3). On review prior to cycle 2, a dramatic clinical response was seen (Figure 4). The patient remained stable and asymptomatic from a respiratory point of view.

## Discussion

Cutaneous metastases from lung cancers are rare. Whilst the most frequent source of cutaneous metastases in men is from the lungs (24%), in women, it’s the least at 4%. Moreover, when present they are commonly found on the chest, abdomen, or back. Clinically and morphologically, they lack a distinctive presentation. They can be multiple, painless, firm, or mobile nodules with normal skin. They are described either as nodular, popular, or zosteriform with an isolated fungating appearance thought to be very seldom in nature. The size can vary from 2 mm to 6 cm [3].

The challenging aspect of this case was that the patient did not report any respiratory signs or symptoms. Furthermore, this case was highly atypical as the patient, a female ex-smoker of 20 years, initially presented with a painless cystic nodule, which then rapidly grew to a large (10 cm), painful, and fungating ulcerating lesion.

## Conclusions

This case illustrates how lung cancer can present with cutaneous metastasis without preceding bronchopulmonary symptoms. It is therefore imperative for clinicians to have a high index of suspicion for lung cancer in active and ex-smokers with a suspicious skin lesion irrespective of the presence of any respiratory symptoms. Similarly, workup must be considered for any high-risk patients when presenting with a suspicious skin lesion due to its lack of distinctive presentation.
